# Feasibility of administration of calcitonin gene-related peptide receptor antagonist on attenuation of pain and progression in osteoarthritis

**DOI:** 10.1038/s41598-023-42673-2

**Published:** 2023-09-16

**Authors:** Akinori Nekomoto, Tomoyuki Nakasa, Yasunari Ikuta, Chenyang Ding, Shigeru Miyaki, Nobuo Adachi

**Affiliations:** 1 Department of Orthopaedic Surgery, Graduate School of Biomedical and Health Sciences, Hiroshima University, 1-2-3 Kasumi, Minamiku, Hiroshima, Hiroshima 734-8551 Japan; 2 Medical Center for Translational and Clinical Research, Hiroshima University Hospital, Hiroshima, Japan

**Keywords:** Experimental models of disease, Translational research

## Abstract

Suppressing inflammation and abnormal subchondral bone turnover is essential for reducing osteoarthritis (OA) progression and pain relief. This study focused on calcitonin gene-related peptide (CGRP), which is involved in inflammation and bone metabolism, and investigated whether a CGRP receptor antagonist (rimegepant) could suppress OA progression and relieve pain in two OA models. C57BL/6 mice (10-week-old) underwent surgical destabilization of the medial meniscus, and Rimegepant (1.0 mg/kg/100 μL) or phosphate-buffered saline (100 μL) was administered weekly intraperitoneally after OA surgery and evaluated at 4, 8, and 12 weeks. In the senescence-accelerated mice (SAM)-prone 8 (SAMP8), rimegepant was administered weekly before and after subchondral bone sclerosis and sacrificed at 9 and 23 weeks, respectively. Behavioral assessment and immunohistochemical staining (CGRP) of the dorsal root ganglion (DRG) were conducted to assess pain. In DMM mice, synovitis, cartilage degeneration, and osteosclerosis were significantly suppressed in the rimegepant group. In SAMP8, synovitis, cartilage degeneration, and osteosclerosis were significantly suppressed by rimegepant at 9 weeks; however, not at 23 weeks. Behavioral assessment shows the traveled distance and the number of standings in the rimegepant group were significantly longer and higher. In addition, CGRP expression of the DRG was significantly lower in the rimegepant group at 8 and 12 weeks of DMM and 9 weeks of SAMP8 treatment. No adverse effects were observed in either of the mouse models. Inhibition of CGRP signaling has the potential to be a therapeutic target to prevent OA progression and suppress pain through the attenuation of subchondral bone sclerosis and synovitis.

## Introduction

Osteoarthritis (OA) causes progressive degeneration of cartilage, subchondral bone, and other joint tissues, resulting in pain and functional disability^1,2^. The exact mechanisms of OA have yet to be fully elucidated, except for obvious risk factors such as age and obesity^2,3^. Since subchondral bone plays an essential role in cartilage metabolism, significant attention has recently been paid to the remodeling of subchondral bone during OA progression^4,5^. Neuropeptides, including substance P, calcitonin gene-related peptide (CGRP), and vasoactive intestinal polypeptide, regulate nociception and local bone metabolism, inflammation, angiogenesis, and cell proliferation^6^. Nerve fibers containing CGRP directly regulate bone remodeling because CGRP promotes osteogenesis and inhibits osteoclastogenesis^7,8^. Numerous studies have reported elevated CGRP levels in patients with OA's serum, synovial fluid, and synovial tissues^9,10^. Pain intensity is also positively associated with serum CGRP levels. Based on previous reports, OA progresses when many CGRP-positive nerve fibers appear in the subchondral bone, and CGRP is expressed in many nerves innervating the subchondral bone^10,11^. Targeting CGRP as a therapeutic target could be an effective way to inhibit OA progression and provide an analgesic effect.

A previous report showed that administering a single dose of the CGRP receptor antagonist, BIBN4096, could ameliorate OA progression by inhibiting subchondral bone sclerosis^12^. Another report demonstrated that CGRP-knockout mice exhibited suppression of OA in the early phase^13^. Concerning OA-related pain, CGRP-neutralizing antibodies alleviated pain^14^. This evidence provides hope for new OA treatment options targeting CGRP. Moreover, the treatment strategy for migraine involving therapeutic targeting of CGRP, and several therapeutic agents have been clinically applied. Therefore, our hypothesis was that if a CGRP receptor antagonist, rimegepant, is already in clinical use for migraine and can be applied to OA treatment through drug repositioning, it could promptly lead to effective treatment for OA. Moreover, using rimegepant will reduce the development cost of a new drug and ensure safety.

This study aimed to examine the feasibility of rimegepant administration, a CGRP receptor antagonist already used in clinical practice for migraine, for OA treatment by investigating whether weekly administration of rimegepant could ameliorate OA progression and pain without adverse effects.

## Methods

### Animal models

Two different OA mouse models were used: trauma-related OA and spontaneous OA. First, C57BL/6 mice (10-week-old male; n = 60) underwent surgical destabilization of the medial meniscus (DMM) as trauma-related OA. According to a previous report, the medial meniscotibial ligament of the right knee joint was resected. Second, intraperitoneal injection of 1.0 mg/kg/100 μL of CGRP receptor antagonist (rimegepant; BMS-927711, Cayman Chemical, MI, USA) was administered immediately after the surgery, followed by regular weekly intraperitoneal injections^8,15,16^. In the control group, phosphate-buffered saline (PBS) was injected after surgery at a dose of 100 µL/animal, followed by regular weekly intraperitoneal injections. Mice were euthanized by anaesthetic overdose of isoflurane at 4, 8, and 12 weeks after surgery and knee joints (each group 4 weeks: n = 6, 8 weeks: n = 6, 12 weeks: n = 18) were harvested. In addition, the sham operation, in which the skin of the right knee joint was cut, was performed on six male mice of the same age as mice in the sham group. Intraperitoneal injection of rimegepant was conducted in the same dosing regimen described above after the operation. The mice were sacrificed in the same manner as other mice at 12 weeks, and the knee joints were harvested.

As a spontaneous age-related OA model, senescence-accelerated mice (SAM)-prone 8 (SAMP8) (4-week- old male; n = 48) were used^17–19^. Mice were obtained from Japan SLC (Shizuoka, Japan). To evaluate the effect of rimegepant before and after subchondral bone sclerosis, two groups were set up, one administered starting at 4 weeks of age before the subchondral bone sclerosis occurs and the other at 13 weeks of age when the subchondral bone sclerosis had already occurred according to the previous report (each group: 4 weeks of age, n = 12; 13 weeks of age, n = 12) (Supplementary Fig. 1)^17,20^. Rimegepant or PBS was administered weekly intraperitoneally, and the group injected at 4 weeks was euthanized at 9 weeks, and the group injected at 13 weeks was euthanized at 23 weeks by an anaesthetic overdose of isoflurane, and tissues were harvested.

In line with a previous report, all mice were housed in groups of three to five per cage (S 143 mm × 293 mm × H 148 mm) with sterilized beta-chip bedding and maintained at 23 ± 1 °C with a 12-h light/dark cycle and acidified water and complete commercial pelleted food ad libitum^17^.

### Micro-computed tomography

The samples were analyzed under high-resolution μ-CT (Skyscan 1176, Bruker, Billerica, MA, USA) using the following parameters: source voltage, 40 kV; source current, 580 μA; pixel size, 18 μm; and spatial resolution, 9 μm (NRecon Version:1.7.4.6, Bruker, Billerica, MA, USA), reconstructed with a multi-planar reconstruction set to any slice angle (Data viewer Version:1.5.6.2, Bruker, Billerica, MA, USA), and analyzed using analysis software (CT An Version:1.20.8.0+, Bruker, Billerica, MA, USA). The thoracolumbar vertebrae below the 13th thoracic vertebra were harvested at sacrifice, and the entire length of the femur, including the knee joint and thoracolumbar vertebrae, was imaged. The ratio of bone volume (BV) to total bone volume (TV) (BV/TV, %) at the subchondral bone of the tibial plateau was measured at 4, 8, and 12 weeks in DMM mice and 9 and 23 weeks in SAMP8 as previously described^21^. The bone mineral density (BMD) of the trabecular bone in the distal femur and third lumbar vertebra (L3) were also measured and calculated in 12-week samples from DMM mice and 23-week samples from SAMP8 based on a previous report^22^.

### Open-field tests

Open-field tests were performed on DMM mice. The tests were performed for each mouse in a rectangular box (37.5 cm length, 42 cm width, 30 cm depth), just before surgery and before sacrifice, based on a previous experiment^23,24^. Each mouse was placed in one corner of an open-field box and was allowed to explore the box freely. The movements of the mice were monitored and recorded for 6 min. In addition, the total distance the mice traveled in the box was calculated using specific devices (SMART, Panlab SL, Barcelona, Spain), and the number of standing was measured.

### Histological analysis

For histological analysis, 4.5-μm-thick sagittal sections for DMM mice and coronal sections for SAMP8 were prepared and stained using safranin-O fast green and hematoxylin–eosin (HE). Pathological changes in the medial femoral condyle and tibial plateau were scored using the Osteoarthritis Research Society International (OARSI) recommendations for OA knee cartilage scoring in mice^12,25^. OA grade was calculated by summing the femur and tibia scores. Synovitis was evaluated at 4 and 12 weeks in the DMM mice and 9 weeks in the SAMP8 using the established synovitis score for changes in synovial lining thickness and cellular density in the synovial stroma (0–3 points, maximum score: 6 points)^26^. As cartilage degeneration was not obvious after 9 weeks of SAMP8, the subchondral bone scoring system reported by Nagira et al. was used^20^. The subchondral bone scoring system has three parameters: the subchondral bone plate consisting of a combination of subchondral bone plate thickness and angiogenesis, BV/TV, and osteophytes in the horizontal plane. The thickness of the subchondral bone plate and BV/TV was measured using ImageJ 1.53a (National Institution of Health, Bethesda, MD, USA) according to previous reports^20,27^. After quantifying each parameter, except for osteophytes, they were graded on a scale of subchondral bone plate thickness 0–6, BV/TV 0–3, and osteophyte 0–3.

### Immunohistochemical analysis

For immunohistochemical analysis, first, each section was immunostained using rabbit anti-CGRP antibody (1:500, ab139264, Abcam, Cambridge, UK), anti-osteocalcin (1:100 dilution, Santa Cruz Biotechnology, Dallas, TX), anti-type 10 collagen (1:15 dilution, The Developmental Studies Hybridoma Bank, IA), and anti-CD31 antibody (1:2000 dilution, ab182981, Abcam, Cambridge, UK) using the 3,30-diaminobenzidine substrate, as described previously^28^. In addition, IgG isotype control was used as a negative control. Second, the sections were incubated with secondary antibodies for 1 h in the dark. The secondary antibody (1:500) for CGRP was Alexa Fluor 488-conjugated anti-rabbit IgG. The secondary antibody (1:500) for CD31 was Alexa Fluor 568-conjugated anti-rabbit IgG. A DAPI (Dojindo Laboratories, Kumamoto, Japan) solution was used for nuclear staining. Immunostaining for a disintegrin and metalloproteinase with thrombospondin motifs 5 (ADAMTS-5) for the evaluation of the expression of aggrecanase and matrix metalloproteinase 13 (MMP13) for evaluation of the expression of the matrix metalloprotease as a key factor of the OA development was also performed^29–31^. Slides were pretreated with antigen-retrieval reagent (Immunoactive; Matsunami Glass Ind, Ltd., Osaka, Japan) at 60 °C for 16 h, followed by blocking serum for 30 min. Third, the sections were immunostained with anti-ADAMTS-5 antibody (GeneTex, GTX100332, 10 µg/mL) and anti-MMP13 antibody (ThermoFisher Scientific, MA5-14328, 20 µg/mL) diluted in Can Get Signal immunostaining solution (TOYOBO, Tokyo, Japan) using a Vectastain ABC-AP alkaline phosphatase kit and AP substrate kit (Vector Laboratories, Burlingame, CA, USA) according to the manufacturer's protocol. The immunohistochemical signals were quantified as follows: For CGRP expression, three rectangle areas (210 × 160 μm) were set in the bone marrow cavity in the subchondral bone epiphysis, and the CGRP positive area in each rectangle area was measured at 200× magnification using ImageJ software (National Institution of Health). The percentage of the total area of each field and the CGRP-positive area were calculated. For the expression of type 10 collagen, MMP13, and ADAMTS-5, three 100 μm × 100 μm squares were randomly set in the cartilage layer, the total cell number and the number of type 10 collagen-positive cells, MMP13-positive cells, and ADAMTS-5 positive cells were counted respectively. Then, the percentage of the positive cells were calculated.

Tartrate-resistant acid phosphatase (TRAP) staining was performed to evaluate osteoclasts using a commercially available kit (Wako Pure Chemical Industries, Ltd., Osaka, Japan) according to the manufacturer's protocol. TRAP-positive multinucleated cells with more than three nuclei were identified as osteoclasts. Osteoclasts in the subchondral bone were counted and corrected by unit area (0.04 mm^2^) to analyze osteoclasts.

In both DMM and SAMP8, 10 µL of 2% Fluoro-gold (FG) (Fluorochrome Inc., Denver, CO), a neuronal retrograde tracer, was injected into the knee joints 1 week before sacrifice^24,28^. At sacrifice, right lumbar dorsal root ganglion (DRG) L3 and L4 were harvested and quickly embedded in Tissue Freezing medium (Triangle Biomedical Sciences, Durham, NC, USA), snap-frozen in liquid nitrogen, and stored at – 80 ℃. Tissues embedded in the compound were serially sectioned into 6-µm sections, and immunohistochemistry for CGRP was performed. Sections of DRGs were incubated with rabbit anti-CGRP (1:500; Abcam, Cambridge, MA) overnight at 4 °C, and the second detection was performed with Alexa Fluor 568-conjugated goat anti-rabbit secondary antibody (1:500 dilution) for 1 h at a temperature of 25 °C. CGRP-positive nerve fibers and FG-positive fibers were counted, and the ratio of the number of CGRP- and FG-positive cells to the number of FG-positive cells was determined using ImageJ software (National Institute of Health). Serial three sections were evaluated, and the average values were calculated.

### Blood examinations

First, at sacrifice, blood was collected from the heart under anesthesia by isoflurane (2.5% inspired concentration in 0.21 FiO_2_), and it was left standing at 4 °C for 2 h. Second, the blood was centrifuged (4 °C, 3000 rpm, 30 min) and the serum was obtained. Third, Alanine aminotransferase (ALT) and aspartate aminotransferase (AST) were measured by Oriental Yeast Co. (Tokyo, Japan).

### Statistical analysis

All results are expressed as mean ± standard deviation (SD). Comparisons among the three groups were made using the Kruskal–Wallis H-test, and the Mann–Whitney U test was used to detect the differences between the two groups. For TRAP staining data, Wilcoxon t-tests with Bonferroni correction were used for comparisons within each group. P < 0.05 indicated statistical significance. These statistical analyses were conducted using SPSS for Windows (version 27.0, IBM Corporation, Armonk, NY, USA).

### Ethics approval

All animal experiments were performed following the Guidelines for Animal Experimentation at Hiroshima University and with the approval of the Committee of Research Facilities for Laboratory Animal Sciences, Graduate School of Biomedical Sciences, Hiroshima University (A21-14). This study is reported in accordance with ARRIVE guidelines.

## Results

### Effect of rimegepant on inhibition of OA progression in DMM mice

**Figure 1:**
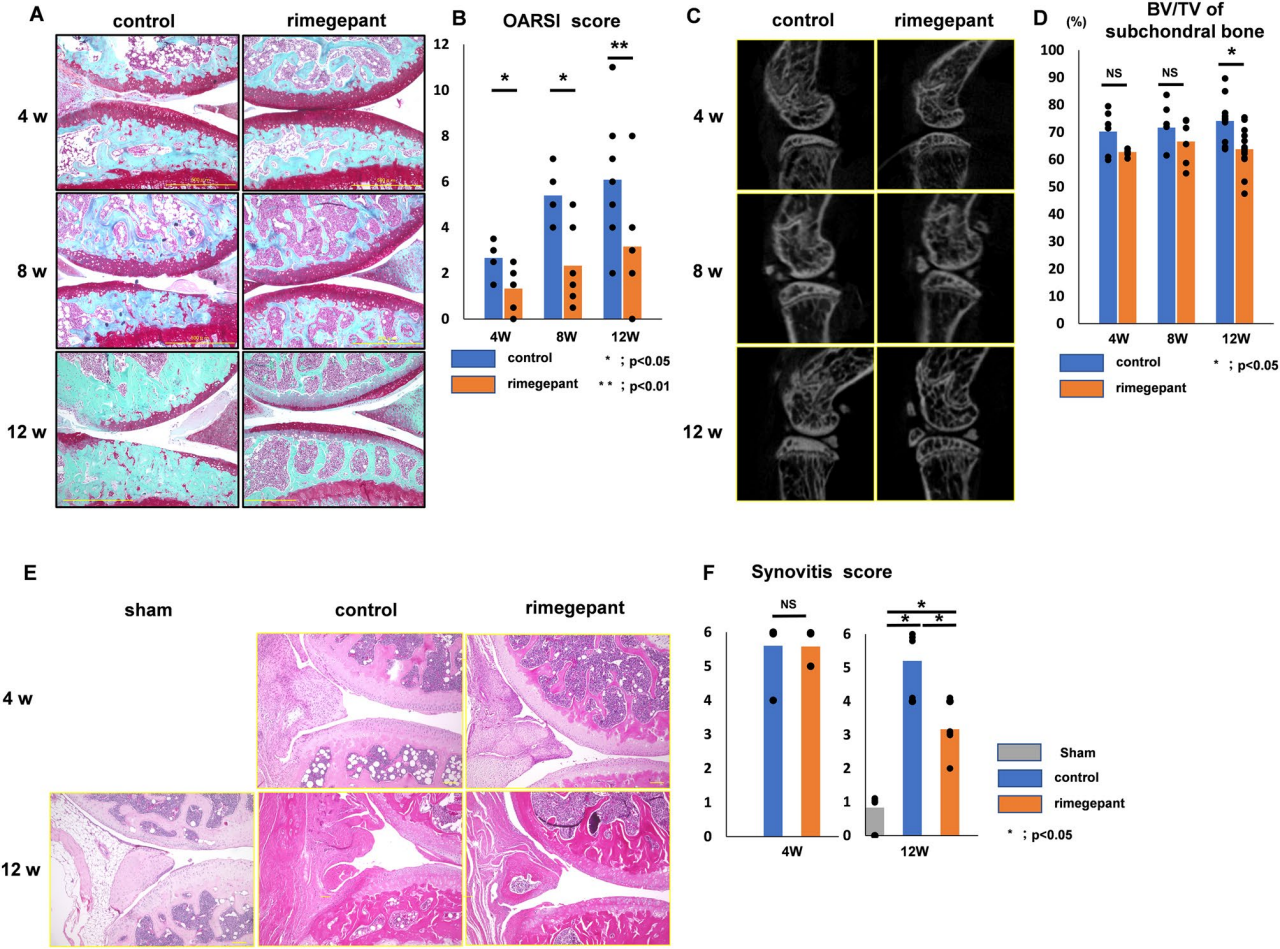
Histological evaluation of DMM mice at 4, 8, and 12 weeks. Histological evaluation of DMM mice at 4, 8, and 12 weeks. (**A**) Safranin O staining of knee joint. The bar indicates 500 µm. (**B**) The OARSI score was obtained by adding the femur and tibia (n = 6 at 4, 8 weeks, n = 12 at 12 weeks for each strain). (**C**) Micro-CT findings. (**D**) BV/TV of the subchondral bone epiphysis (n = 6 at 4, 8 weeks, n = 18 at 12 weeks for each strain). (**E**) HE staining of the knee joint. Bars indicate 100 μm. (**F**) Synovitis scores of DMM and sham mice at 4 and 12 weeks after surgery (n = 6 for each strain). A comparison of mean values was performed using the Kruskal–Wallis H-test. All data except for Synovitis scores were compared between control and rimegepant group by Mann–Whitney U test at each time point. *P < 0.05 and **P < 0.01 were statistically significant.

All the mice survived the experimental procedures without experiencing any adverse effects. There was no difference in body weight between the rimegepant and control groups at time 0 and at 4, 8, and 12 weeks postoperatively. The control group exhibited progressive degenerative OA changes over time. In contrast, rimegepant treatment reduced cartilage degeneration (Fig. 1A). The sum of the OARSI scores for the femur and tibia in the rimegepant group was significantly lower than that in the control group at 4, 8, and 12 weeks (P = 0.045 at 4 weeks, P = 0.044 at 8 weeks, P = 0.0082 at 12 weeks; Fig. 1B). However, in the analysis of µCT images, although there was no significant difference of the BV/TV ratio of the medial plateau's subchondral bone between the two groups at 4 and 8 weeks (P = 0.59 at 4 weeks, P = 0.36 at 8 weeks), BV/TV ratio of that the medial plateau's subchondral bone in the control group was significantly higher than in the rimegepant group at 12 weeks (P = 0.035 at 12 weeks; Fig. 1C and D). In addition, there is no significant difference in the synovitis score at 4 weeks after surgery between the control and the rimegepant groups. However, the synovitis score of the control group was the highest, and that of the sham group was significantly lower than that of the rimegepant group at 12 weeks after surgery (P = 0.0012, Fig. 1E and F).

**Figure 2:**
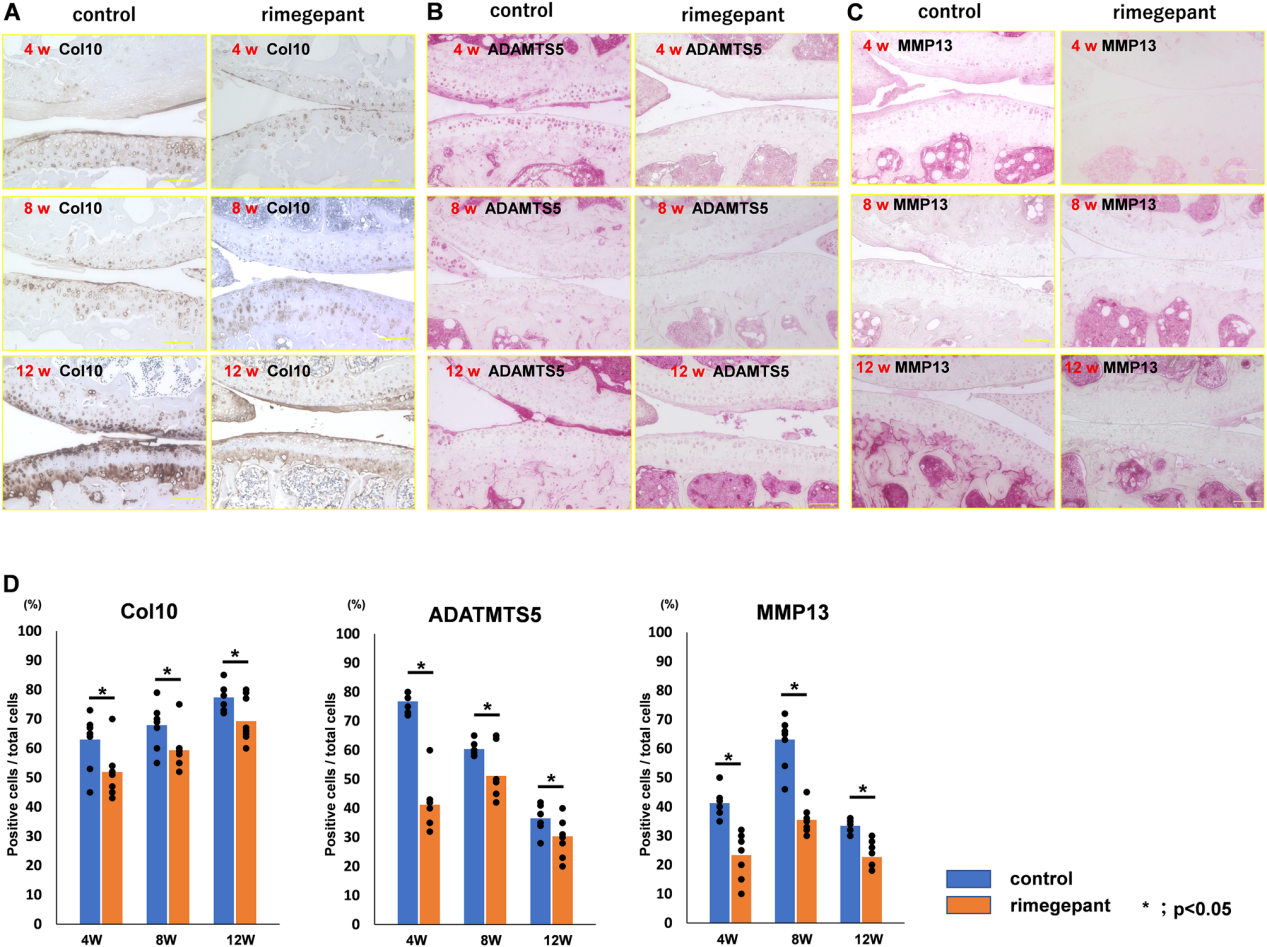
Immunohistochemistry analysis on articular cartilage. (**A**) Immunohistochemistry of Type 10 collagen (Col 10). (**B**) ADAMTS-5. (**C**) MMP 13. The bar indicates 100 μm. (**D**) The ratio of Col 10, MMP13, or ADAMTS-5 positive cells and total cells (n = 6 at 4, 8 weeks, n = 9 at 12 weeks for each strain). All data were compared between control and rimegepant group by Mann–Whitney U test at each time point. *P < 0.05 was statistically significant. ADAMTS-5, a disintegrin and metalloproteinase with thrombospondin motifs 5; MMP13, Matrix metallopeptidase 13.

Immunohistochemistry revealed that rimegepant suppressed the expression of type 10 collagen, MMP13, and ADAMTS-5, while the control group exhibited the expression of type 10 collagen, MMP13, and ADAMTS-5 intensely (Fig. 2A–C). The rate of type 10 collagen, MMP13, and ADAMTS-5 positive cells/total articular cartilage cells in the control group were significantly higher than those in the rimegepant group at 4, 8, and 12 weeks (P = 0.048, P = 0.0022, and P = 0.0022, respectively at 4 weeks, P = 0.041, P = 0.0022, and P = 0.048, respectively at 8 weeks, and P = 0.041, P = 0.0027, and P = 0.048, respectively at 12 weeks; Fig. 2D).

**Figure 3:**
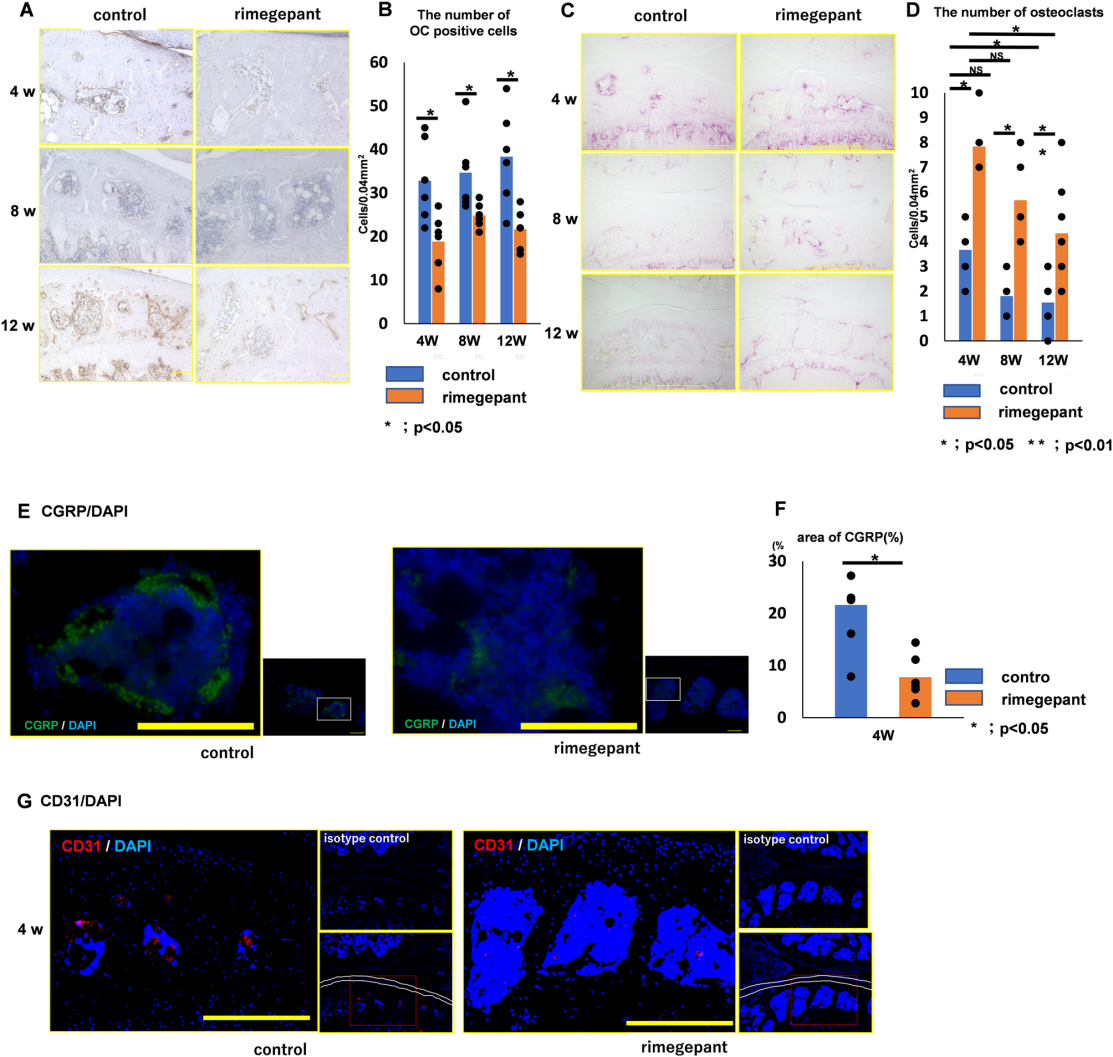
Effects on bone metabolism in the subchondral bone. (**A**) Immunohistochemistry of osteocalcin. The bar indicates 500 μm. (**B**) The number of osteocalcin-positive cells in the subchondral bone's epiphysis (n = 6 at 4, 8, and 12 weeks for each strain). (**C**) TRAP staining. The bar indicates 100 μm. (**D**) The number of TRAP-positive cells in the subchondral bone's epiphysis. (**E**) Immunohistochemistry of CGRP/DAPI in marrow cavity in the subchondral bone. The bar indicates 100 μm. (**F**) Area of CGRP in the subchondral bone's epiphysis at 4 weeks post-surgery. (**G**) Immunohistochemistry of CD31/DAPI in marrow cavity in the subchondral bone. Confirmation of nonspecific staining using isotype control. The area surrounded by two white lines is the cartilage layer. All data except for the data of TRAP staining were compared between control and rimegepant group by Mann–Whitney U test at each time point. *P < 0.05 was statistically significant. For TRAP staining data, Wilcoxon t-test with Bonferroni correction was used to compare between 4 and 8 weeks of age or between 4 and 12 weeks of age within each group, and Mann–Whitney U test was used to compare between control and rimegepant group at each time point. *P < 0.05 and **P < 0.01 were statistically significant. NS, not significant. TRAP, tartrate-resistant acid phosphatase; CGRP, calcitonin gene-related peptide.

The ratio of osteocalcin-positive cells to bone marrow cells in the control group was significantly higher than that in the rimegepant group at 4, 8, and 12 weeks (P = 0.034, P = 0.034, and P = 0.025, respectively; Fig. 3A and B). The number of TRAP-positive cells in the control group was significantly lower than that in the rimegepant group at 4, 8, and 12 weeks (P = 0.015, P = 0.019, and P = 0.0011, respectively; Fig. 3C and D). CGRP expression was observed in the marrow cavity of the subchondral bone, and its expression in the control group was significantly higher than in the rimegepant group at 4 weeks (P = 0.013; Fig. 3E and F). At 8 and 12 weeks, the marrow cavity of the subchondral bone in the control group had disappeared and could not be compared with the rimegepant group. Immunohistochemistry for CD31 resulted in a few vessels being evaluated in the bone marrow cavity, but vascular channels could not be observed at the osteochondral junction (Fig. 3G).

### Pain assessment in the DMM model mice

**Figure 4:**
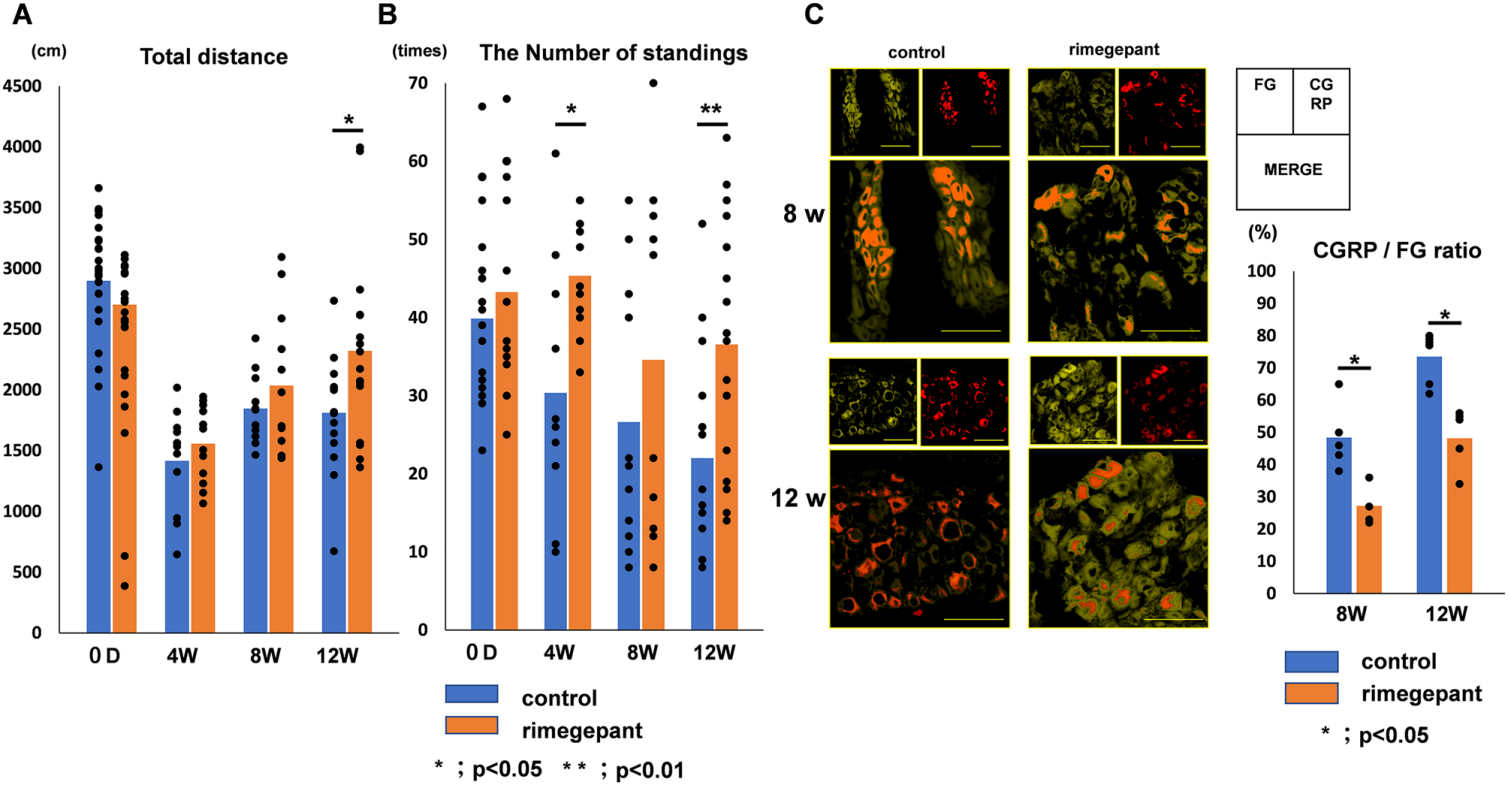
Assessment of the analgesic effect. (**A**) Total distance traveled. (n = 6 at 4, 8 weeks, n = 18 at 12 weeks for each strain). (**B**) The number of standings. (n = 6 at 4, 8 weeks, n = 18 at 12 weeks for each strain). (**C**) Immunofluorescence staining for FG and CGRP of DRG. The CGRP/FG ratio of DRG in the rimegepant group was significantly lower than that in the control at 8 and 12 weeks post-surgery (n = 4–5 at 8 and 12 weeks for each strain). All data were compared between control and rimegepant group by Mann–Whitney U test at each time point. *P < 0.05 was statistically significant. FG, fluoro-gold; CGRP, calcitonin gene-related peptide; DRG, dorsal root ganglion.

Compared with the rimegepant group, the control groups showed significantly reduced total distance traveled in the open field test at 12 weeks (P = 0.027; Fig. 4A). Furthermore, the control group showed a reduced number of times in standing at 4 weeks (P = 0.018) and 12 weeks (P = 0.0077; Fig. 4B). Immunofluorescence staining for CGRP of the DRG showed that the percentage of CGRP- and FG-positive fibers gradually increased over time. The percentage of the rimegepant group at 8 weeks (control group: 48.4 ± 10.3%, rimegepant group: 27.2 ± 6.4%) and 12 weeks (control group: 73.5 ± 7.9%, rimegepant group: 48.1 ± 8.5%) were significantly lower than that of the control group (P = 0.018 at 8 weeks, P = 0.0068 at 12 weeks; Fig. 4C).

### Effect of rimegepant on inhibition of OA progression in SAMP8

**Figure 5:**
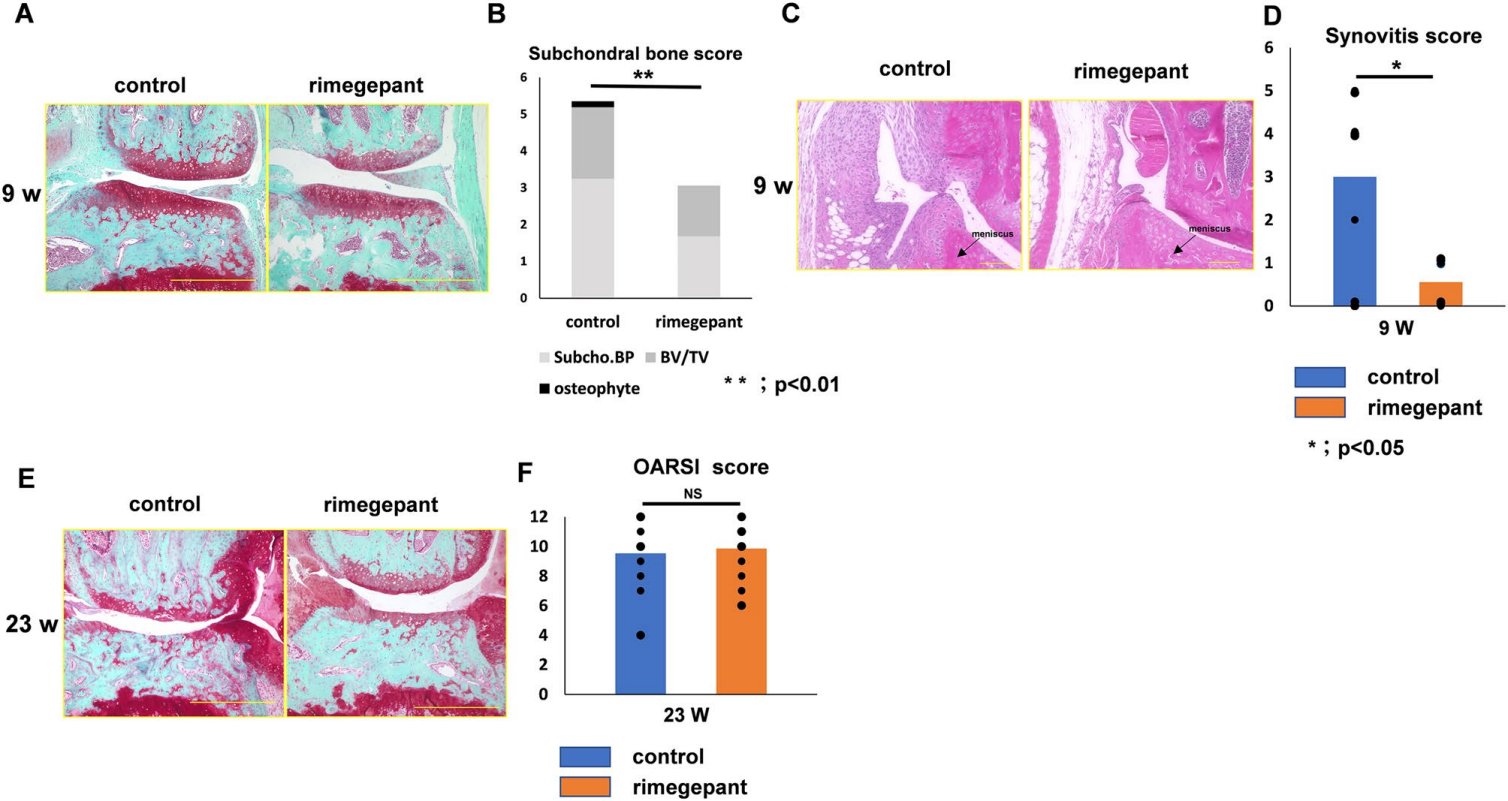
Histological evaluation in SAMP8 at 9 and 23 weeks. (**A**) Safranin O staining of the knee joint at 9 weeks. The bar indicates 500 μm. (**B**) Subchondral bone score (n = 12 for each strain). (**C**) HE staining. The bar indicates 100 μm. (**D**) The synovitis score of SAMP8 at 9 weeks (n = 8 for each strain). (**E**) Safranin O staining of the knee joint at 23 weeks. The bar indicates 500 μm. (**F**) OARSI score at 23 weeks. (n = 12 for each strain). All data were compared between control and rimegepant group by Mann–Whitney U test at each time point. *P < 0.05 and **P < 0.01 were statistically significant. SAMP8, senescence-accelerated mice-prone 8; NS, not significant.

Further examination was conducted on the effect of rimegepant in SAMP8 as a spontaneous OA model^17–19^. All the mice survived the experimental procedures without experiencing any adverse effects. There was no difference in body weight between the rimegepant and control groups at 4 and 9 weeks of age and 13 and 23 weeks of age. No loss of staining in the articular cartilage was observed in either group at the 9-week (Fig. 5A). In contrast, the subchondral bone score was significantly higher in the control group (control group: 5.35 ± 1.47, rimegepant group: 3.06 ± 2.05, P = 0.0368; Fig. 5B). A certain degree of synovitis was observed in the control group (Fig. 5C), and the score was also significantly higher in the control group (control group: 3.0 ± 2.1, rimegepant group: 0.6 ± 0.5) (P = 0.049; Fig. 5D). The sum of the OARSI score in the 23 weeks model was not statistically different between the two groups (control group: 9.5 ± 2.2, rimegepant group: 9.8 ± 1.8) (Fig. 5E and F).

**Figure 6:**
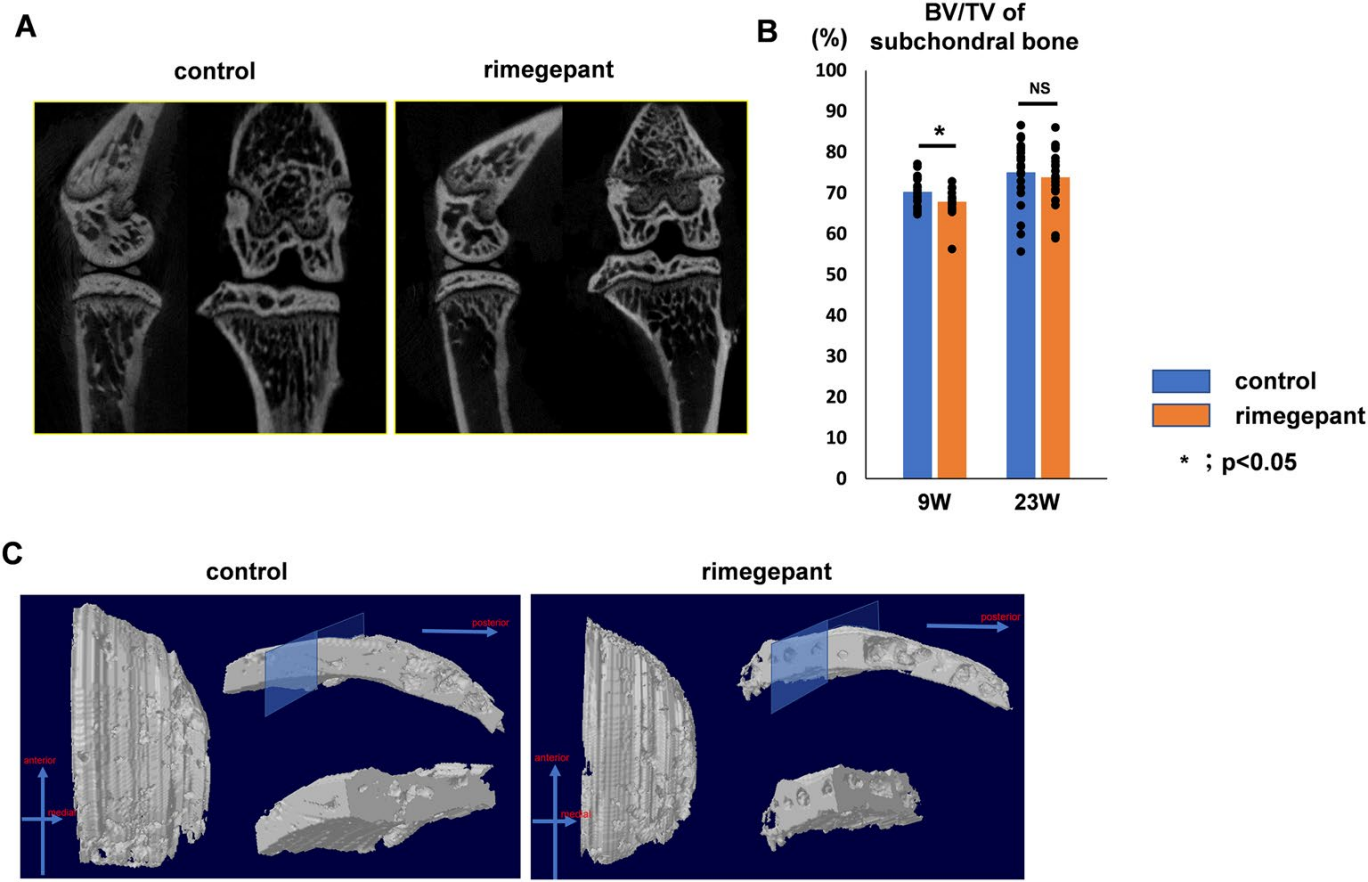
Bone morphological assessment of SAMP8 using μCT. (**A**) μ-CT coronal images of the knee joint at 9 weeks. In the control group, bone sclerosis is particularly prominent on the medial tibial plateau, but in the rimegepant group, bone sclerosis is less common. (**B**) The BV/TV of subchondral bone at 9 weeks and 23 weeks. (n = 12 for each strain. Left and right knees were measured, and the average was calculated as the individual data.). All data were compared between control and rimegepant group by Mann–Whitney U test at each time point. *P < 0.05 was statistically significant. (**C**) Cross-Sectional observation with 3DCT. BV, bone volume; TV, total bone volume; 3DCT, 3 dimensional computer tomography.

Analysis of µCT images showed that in the 9 weeks model, the BV/TV ratio of the medial plateau's subchondral bone in the control group was significantly higher than that in the rimegepant group (control group: 71.9 ± 3.0, rimegepant group: 67.0 ± 2.7) (Fig. 6A, P = 0.044; Fig. 6B). There was no significant difference between the two groups in the 23 weeks model (control group: 75.0 ± 8.0, rimegepant group: 73.8 ± 6.6). (Fig. 6B). On three dimensional computer tomography (3DCT), it was also observed that the control group had more osteosclerosis of the subchondral bone than the rimegepant group (Fig. 6C).

**Figure 7:**
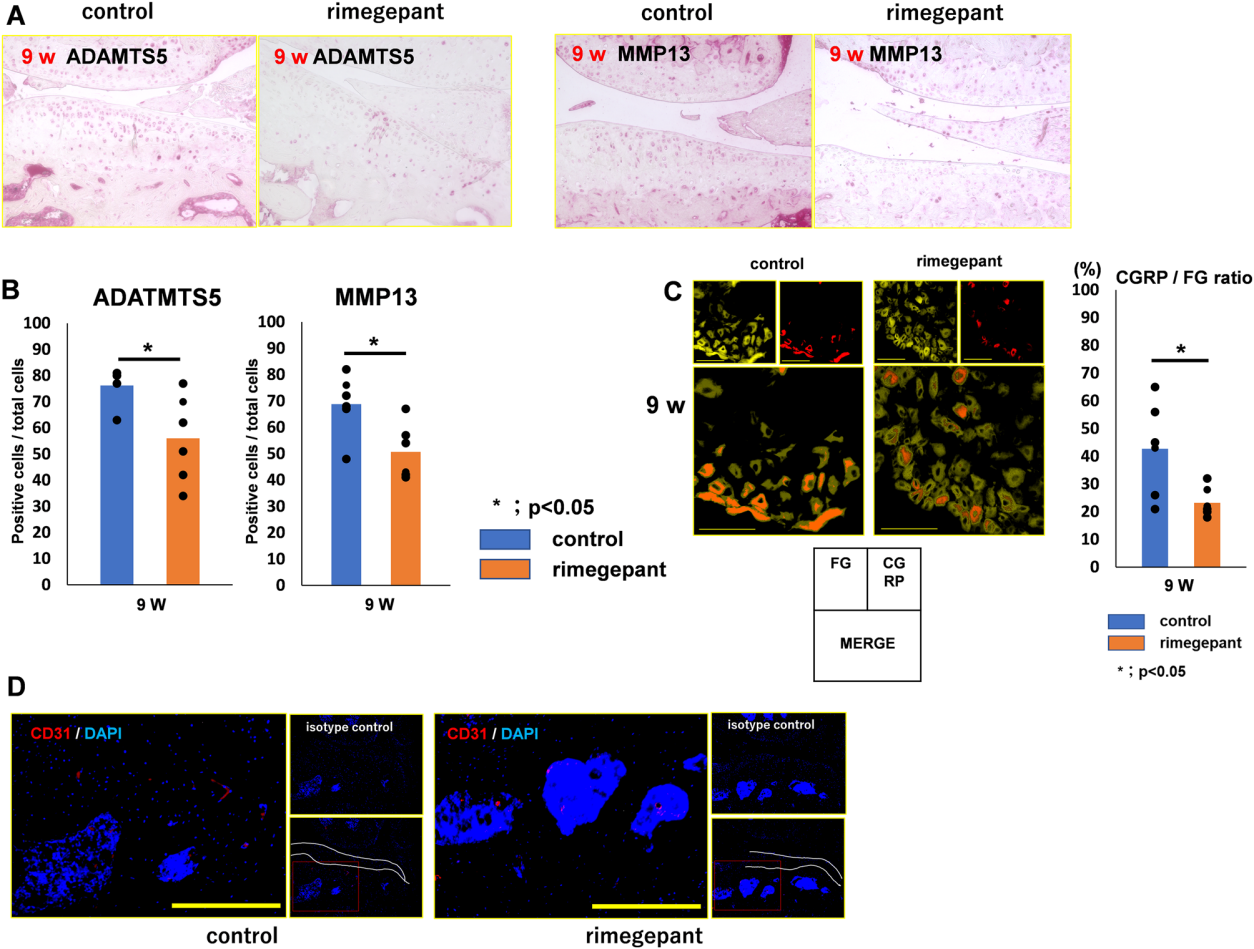
Evaluation of articular cartilage and DRG in SAMP8 at 9 weeks. (**A**) Immunohistochemistry of ADAMTS-5 and MMP 13. (**B**) The ratio of MMP13 or ADAMTS-5 positive cells and total cells (n = 6 for each strain). (**C**) Immunofluorescence staining for FG and CGRP of DRG. CGRP/FG ratio in the rimegepant group was significantly lower than that in the control at 9 weeks (n = 6 for each strain). (**D**) Immunohistochemistry of CD31/DAPI in marrow cavity in the subchondral bone. Confirmation of nonspecific staining using iso type control. The area surrounded by two white lines is the cartilage layer. The bar indicates 100 μm. All data were compared between control and rimegepant group by Mann–Whitney U test at each time point. *P < 0.05 was statistically significant. ADAMTS-5, a disintegrin and metalloproteinase with thrombospondin motifs 5; MMP13, Matrix metallopeptidase 13; CGRP, calcitonin gene-related peptide; DRG, dorsal root ganglion; FG, fluoro-gold; NS, not significant.

Immunohistochemistry in the 9 weeks model revealed that rimegepant suppressed the expression of MMP13 and ADAMTS-5. In contrast, the control group exhibited intense expression of MMP13, and ADAMTS-5 (Fig. 7A). The rate of MMP13 and ADAMTS-5 positive cells/total cells in articular cartilage in the control group was significantly higher than that in the rimegepant group (P = 0.044 and P = 0.039, respectively; Fig. 7B). However, the 23-weeks model could not be evaluated because of severe cartilage destruction.

Immunohistochemistry for CD31 was performed in the 9 weeks model. Similar to the results of immunohistochemistry for CD31 in the DMM models at 4 weeks postoperatively, vascular channels could not be observed at the osteochondral junction (Fig. 7D).

### Assessment of immunofluorescence staining for CGRP of the DRG in SAMP8

For immunofluorescence staining for CGRP of the DRG, the percentage of CGRP- and FG-positive fibers was calculated. In the SAMP8 in the 9 weeks model, the percentage of CGRP and FG positive fibers in the control group was higher than that in the Rimegepant group (control group: 42.6 ± 10.2%, rimegepant group: 23.2 ± 6.4%, P = 0.023; Fig. 7C).

### Side effect of rimegepant administration on osteoporosis and liver damage

**Figure 8:**
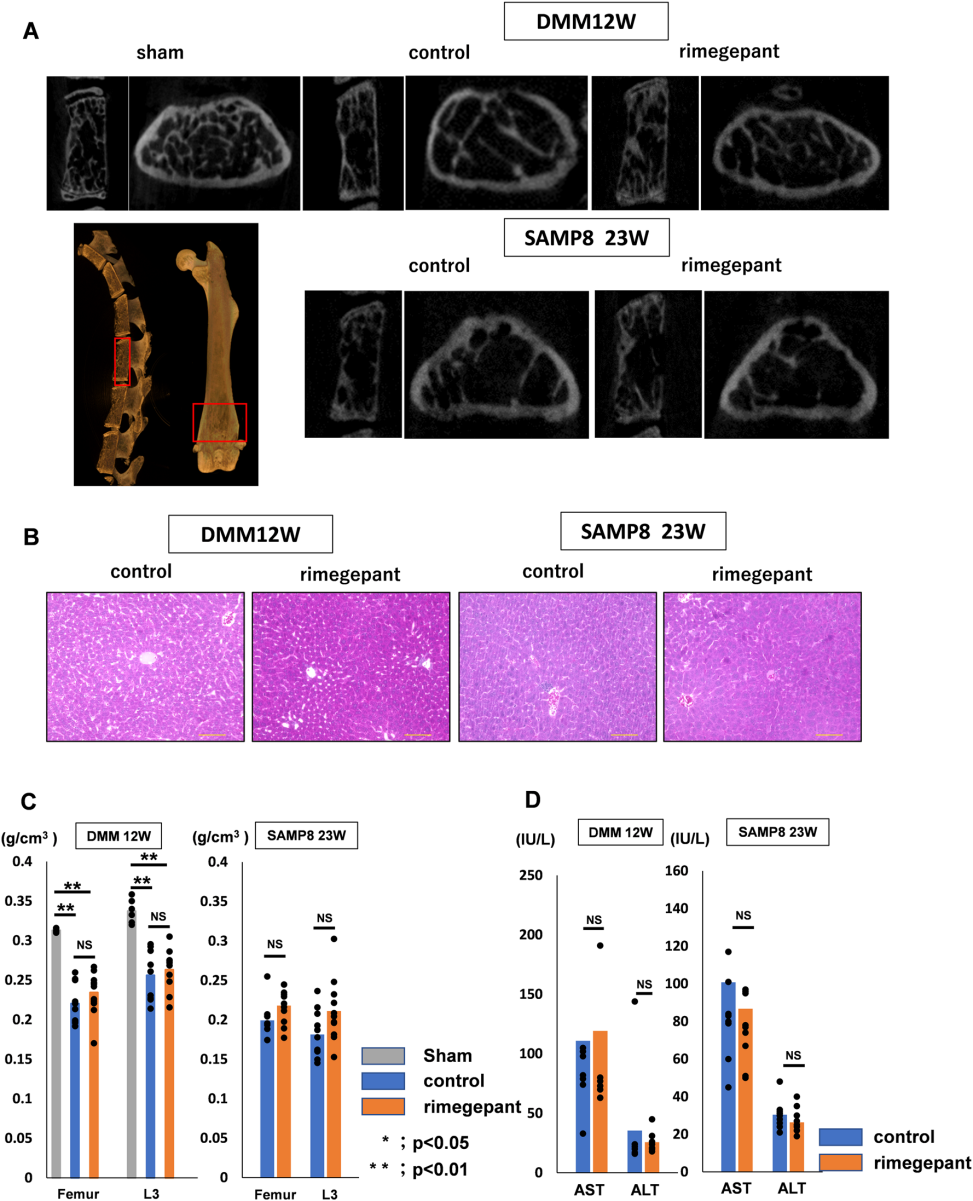
Assessment of the adverse event on bone and liver. (**A**) μ-CT slices of the sagittal view of the 3rd lumbar vertebra and the axial view of the distal femur in the DMM mice and sham mice at 12 weeks and the SAMP8 model at 23 weeks. (**B**) HE staining of liver sections. There was no apparent infiltration of inflammatory cells. (**C**) BMD of the distal femur and L3. In the DMM mice, comparison of mean values was performed using Kruskal Wallis H-test (sham, n = 6; n = 12 for each strain). In the SAMP8, comparison of mean values was performed using Mann–Whitney U test at each time point (n = 12 for each strain). *P < 0.01 was statistically significant. (**D**) AST and ALT serum level. There were no statistically significant differences in AST and ALT in either the DMM or SAMP8 models. DMM, destabilization of the medial meniscus; SAMP8, senescence-accelerated mice prone 8; BMD, bone mineral density; AST; aspartate aminotransferase; ALT; aminotransferase.

BMD was measured in the distal femur and third lumbar vertebra (Fig. 8A). There was no significant difference in BMD between the rimegepant group (femur: 0.23 ± 0.03 g/cm^3^, L3: 0.25 ± 0.03 g/cm^3^) and the control group (femur: 0.22 ± 0.02 g/cm^3^, L3: 0.25 ± 0.03 g/cm^3^), the BMD of the two groups was significantly lower than that of the sham-operated rimegepant group (femur: 0.31 ± 0.02 g/cm^3^, L3: 0.33 ± 0.02 g/cm^3^, P = 0.00027;Fig. 8C). In SAMP8, there was no difference between the two groups in the femur (rimegepant group: 0.22 ± 0.02 g/cm^3^, control group: 0.19 ± 0.02 g/cm^3^) and in the third lumbar spine (rimegepant group: 0.21 ± 0.04 g/cm^3^, control group: 0.18 ± 0.03 g/cm^3^). Liver sections were evaluated, and AST (IU/L) and ALT (IU/L) were measured in serum samples to evaluate liver damage. In addition, liver tissue sections were evaluated by HE staining. Neither the 12-week DMM model nor the 23-week SAMP8 model showed inflammatory cell infiltration or fibrosis in the lobules, as observed in drug-induced liver injury (Fig. 8B).

The deleterious enzymes AST (IU/L) and ALT (IU/L), in hepatocytes, were as follows: AST (rimegepant group, 119 ± 97 IU/L; control group, 110 ± 85 IU/L) and ALT (rimegepant group: 30 ± 20 IU/L. Control group: 42 ± 46 IU/L) in the DMM model; AST (rimegepant group: 86 ± 38 IU/L, control group: 100 ± 48 IU/L) and ALT (rimegepant group: 26 ± 6.5 IU/L, control group: 31 ± 7.2 IU/L) in SAMP8. There were no significant differences in AST and ALT levels between the rimegepant and control groups (Fig. 8D).

## Discussion

The current study demonstrated the feasibility of rimegepant administration to ameliorate OA progression without any adverse effects in two different mice models and OA-related pain in the DMM mice model. As rimegepant has already been used for migraine, it is speculated to exhibit similar results in human OA as in our mice models.

Inflammation and changes in subchondral bone in OA are associated with cartilage degeneration and pain. The condition of the subchondral bone is essential for maintaining cartilage homeostasis^5^. Abnormal mechanical loading leads to increased subchondral bone turnover, whereas angiogenesis and nerve innervation facilitate chondrocyte hypertrophy and cartilage degradation^32^. Synovitis also plays an important role in the pathogenesis of OA. It is correlated with the severity of OA, clinical symptoms, and cartilage degradation by impairing the drainage function of the synovial lymphatic system^33^. Treatment to reduce inflammation and turnover of subchondral bone is a potential strategy for pain relief^34^. The administration of bisphosphonates such as zoledronate to an OA model alleviated arthralgia by suppressing osteosclerosis of the subchondral bone^35^. We focused on CGRP because it plays a crucial role in bone metabolism by inhibiting osteoclast differentiation and promoting osteoblast activity, in addition to affecting peripheral sensitization, inflammation, and pain^36–38^. CGRP acts on various cells, such as vascular endothelial cells, intra-articular synovium, and osteoclasts. It also acts in an autocrine fashion on the released nerve itself, further increasing sensitization and neurogenic inflammation, which is said to have various effects, including pain transmission^39,40^. Concerning the effect of CGRP inhibition on OA, the DMM model using CGRP knockout mice showed suppression of OA in the early phase^13^. It is reported that administration of the olcegepant, CGRP receptor antagonist, for the DMM mice ameliorates OA progression by inhibiting subchondral bone sclerosis^12^. The results of these in *vivo* studies are supported by numerous in vitro studies on bone metabolism of CGRP. CGRP has been shown to promote osteoblast proliferation and differentiation of bone marrow stromal cells by stimulating canonical Wnt signaling and inhibiting osteoblast apoptosis^41,42^. CGRP signaling maintains bone mass for bone resorption by directly stimulating stromal cell osteoblastic differentiation and inhibiting receptor activators of nuclear factor kappa-B ligand-induced nuclear factor kappa B activation, osteoclastogenesis, and bone resorption^43,44^. In addition to bone metabolism, it has been reported that targeting CGRP could suppress pain and inflammation. A CGRP receptor antagonist normalized the enhanced mechanical-evoked responses of joint nociceptors in OA models in rats^45^. In CGRP gene-modified mice, inflammatory pain decreases in the CGRP knockout mouse^46^, and hyperalgesia occurs in the CGRP transgenic mouse^47^. It is well known that synovitis is progressed by angiogenesis and CGRP, which are powerful angiogenetic factors expressed in synovitis^10,48^. CGRP has pro-angiogenic effects in vivo and in vitro, and CGRP receptor antagonists can inhibit angiogenesis (Supplementary Fig. 2)^49^. In this study, we were able to evaluate a few vessels in the bone marrow cavity but could not observe vascular channels at the osteochondral junction as reported using sections from OA patients by Mapp et al.^1^.

That may be due to the difficulty in observing osteochondral junction vessels in mice because of their small diameter and minor number. Although it was difficult to evaluate the inhibitory effect on angiogenesis by rimegepant using immunohistochemistry for CD31 in this study, previous reports regarding osteogenic and angiogenetic effects of CGRP support out results which the administration of rimegepant before the subchondral bone sclerosis could attenuate OA progression by inhibiting osteogenesis and angiogenesis. Sanada et al. showed the OA-related changes and mechanisms in SAMP8, in which osteosclerosis of the subchondral bone occurs from 6-weeks of age and 23 weeks of age exhibited partial or full-thickness cartilage defects with exposure of subchondral bone on a medial tibial plateau with menisci degeneration and osteophytes in all cases^17^. In SAMP8, reducing the Safranin O staining, roughened articular surface, and fibrillations were started at 11 weeks of age. The results using SAMP8 indicated that administration of rimegepant could ameliorate OA progression by inhibiting the subchondral bone sclerosis before obvious degeneration of the articular cartilage, but administration of rimegepant after cartilage degeneration could not ameliorate OA progression because it could not inhibit the progression of cartilage degeneration by the mechanical stress. It is expected that administration of rimegepant ameliorate OA progression and improve OA-related pain in early-stage OA.

Whereas there is a report that CGRP blockade by CGRP antibody (galcanezumab), a humanized monoclonal antibody, was not associated with reductions in signs and symptoms of knee OA in a randomized clinical trial^50^. The reason for the lack of pain relief in that trial can be inferred from our results as follows. The participants in that trial were required to have progressive pain and Kellgren-Lawrence (K-L) grade 2 or 3 on X-ray. They have already occurred osteophyte formation and joint space narrowing. In other words, based on the report regarding SAMP8 by Sanada et al., the participants in that trial have already developed bone sclerosis in the subchondral bone, corresponding to 14 weeks of age in SAMP8^17^. Therefore, the failure to obtain pain relief in that clinical trial may be similar to the results of our failure to suppress OA by starting administration of rimegepant from 13 weeks of age in SAMP8.

There were concerns that serial administration of rimegepant might cause liver damage and osteoporosis, but no adverse effects were observed in this study. Clinical trials for migraine with telcagepant, one of the CGRP receptor antagonists, have been stopped due to liver damage^51^. In contrast, rimegepant does not cause liver damage during migraine use in the clinical setting^52^. Regarding abnormal bone metabolism, previous clinical trials using anti-nerve growth factor (NGF) antibodies showed rapid progressive OA, bone destruction, and osteoporosis^53^. In our study, serial administration of rimegepant did not induce osteoporotic changes because the signaling pathway of CGRP, which is the downstream of NGF, may adjust the bone metabolism moderately by the crosstalk action between two neuropeptides of substance P and CGRP in the control of bone morphogenetic protein 2 signal^54^. As with its use for migraine, drug repositioning of rimegepant to OA would cause few adverse events.

This study had several limitations. First, the blood distribution and concentration of rimegepant were not investigated. Second, in this study, rimegepant was administered once a week, considering that the terminal T1/2 of rimegepant was reported to be 10–12 h in humans^55^. The dosage may be appropriate based on previous reports^52^; however, whether the frequency is appropriate is still unclear. Third, only intraperitoneal administration was performed. The methods of drug administration in OA include intraarticular injection and oral administration. Since commercially available rimegepant is defined as oral administration, its effectiveness in OA should be investigated by oral administration. In addition, it was necessary to show that systemic administration of the rimegepant effectively prevented OA progression and OA-related pain; however, only intraperitoneal administration was chosen. Various methods of administration should be investigated in future studies. Fourth, CGRP and CD31 expression was evaluated in the subchondral bone marrow cavity but in the subchondral bone plate. CGRP and CD31 expression was not clearly observed in the subchondral bone plate in this study. In mice, there is little vascular and neural invasion from the subchondral bone to the cartilage layer. Therefore, it is possible that CGRP and CD31 expression was also not observed. Finally, adverse events were investigated only for osteoporosis and liver disorders. In addition, this study was conducted using mice model, which may differ from that of humans. The safety and effectiveness of rimegepant administration for OA should be evaluated in human clinical trials in the future.

In conclusion, the systemic administration of rimegepant could attenuate OA progression in trauma-induced OA mice models and spontaneous OA mice models before subchondral bone sclerosis. OA-related pain could be attenuated by the administration of rimegepant in trauma-induced OA mice models. Additionally, Rimegepant has already been used clinically for migraine, and drug repositioning of rimegepant could be a safe and effective new treatment strategy for OA.

## Conclusion

### Effect of rimepegant in osteoarthritis

Rimegepant administration ameliorated OA progression in two different mice models and OA-related pain in DMM model mice without any adverse effects. As a possible mechanism, inhibition of CGRP signaling may have prevented the progression of OA and suppressed pain through attenuation of subchondral bone sclerosis and synovitis.

### Supplementary Information

Supplementary Information.

## Data Availability

Datasets analyzed during the current study are available from the corresponding author on reasonable request.
