# Supplementary material for: Feasibility of administration of calcitonin gene-related peptide receptor antagonist on attenuation of pain and progression in osteoarthritis
Source: Sci Rep. 2023 Sep 16;13:15354. doi: 10.1038/s41598-023-42673-2 (PMC10505157; doi:10.1038/s41598-023-42673-2)
Supplement: Supplementary file 1 — Supplementary Information. [file 41598_2023_42673_MOESM1_ESM.docx]

**Supplementary figure 1**

**SafO staining sections before (4 weeks) and after (14 weeks) subchondral bone sclerosis are shown.**

In SAMP8 as spontaneous OA models, rimegepant was administered weekly before and after subchondral bone sclerosis respectively. Nagira et al.[1] and Sanada et al.[2] reported as follows. From 4 to 9 weeks of age, SAMP8 had intact articular cartilage and good proteoglycan staining. At 14 weeks of age, OA severity in SAMP8 ranged from minimal changes, such as reduction of proteoglycan to cartilagefibrillationn or partial defects.

Based on the above, two models were created. One in which intraperitoneal injection of rimegepant was started at 4 weeks, when subchondral bone sclerosis had not yet occurred, and sacrificed at 9 weeks when subchondral bone sclerosis would have been strongly expressed in the natural course. In another model, the intraperitoneal injection of rimegepant was started at 13 weeks after sclerosis of subchondral bone had already occurred, and the animals were sacrificed at 23 weeks. Sections of SafO staining at 4 weeks (Fig.1A) before subchondral bone sclerosis and at 14 weeks (Fig.1B) after subchondral bone sclerosis are shown.


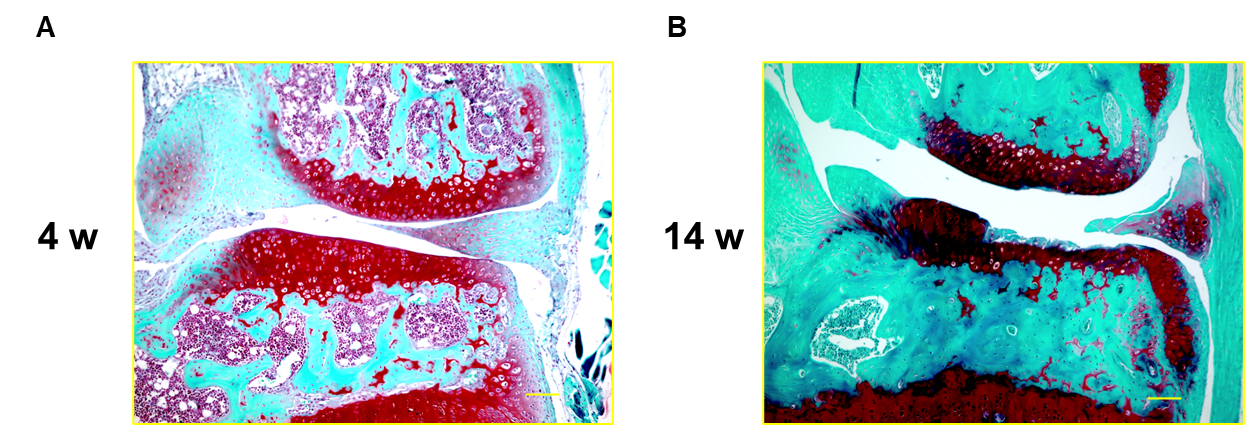


**Figure.1** SafO staining sections (A) 4 weeks. (B) 14 weeks. The bar indicates 100 μm.

**Supplementary figure 2**

**Evaluation of the effect of rimegepant on angiogenesis**

**Material and method**

Human umbilical vein endothelial cells ([HUVECs], Lonza Group AG, Basel, Switzerland) were pre-cultured in an endothelial basal medium-2 (Lonza, Basel, Switzerland) and reseeded at a density of 1.0×104 cells/well in 96-well plates that were pre-coated with Matrigel Matrix (Merck MillipoFre, Billerica, MA). CGRP receptor antagonist (Rimegepant；BMS-927711, Cayman Chemical, Michigan, USA) was added to the wells at doses of 1, 10 and 100 nM, and PBS was added to the control solution. The cells were incubated at 37 C with 5% CO2, and branch points were counted at 8 h after incubation, according to the method previously described[3]. The total tube length was measured using Image J. All experiments were performed at least three times.

**Result**

The branch points in the rimegepant 10 nM group and 100 nM group were significantly lower than in the control group (Fig.2B). The total tube length was significantly shorter in the rimegepant 100 nM than in the control group (Fig.2C).


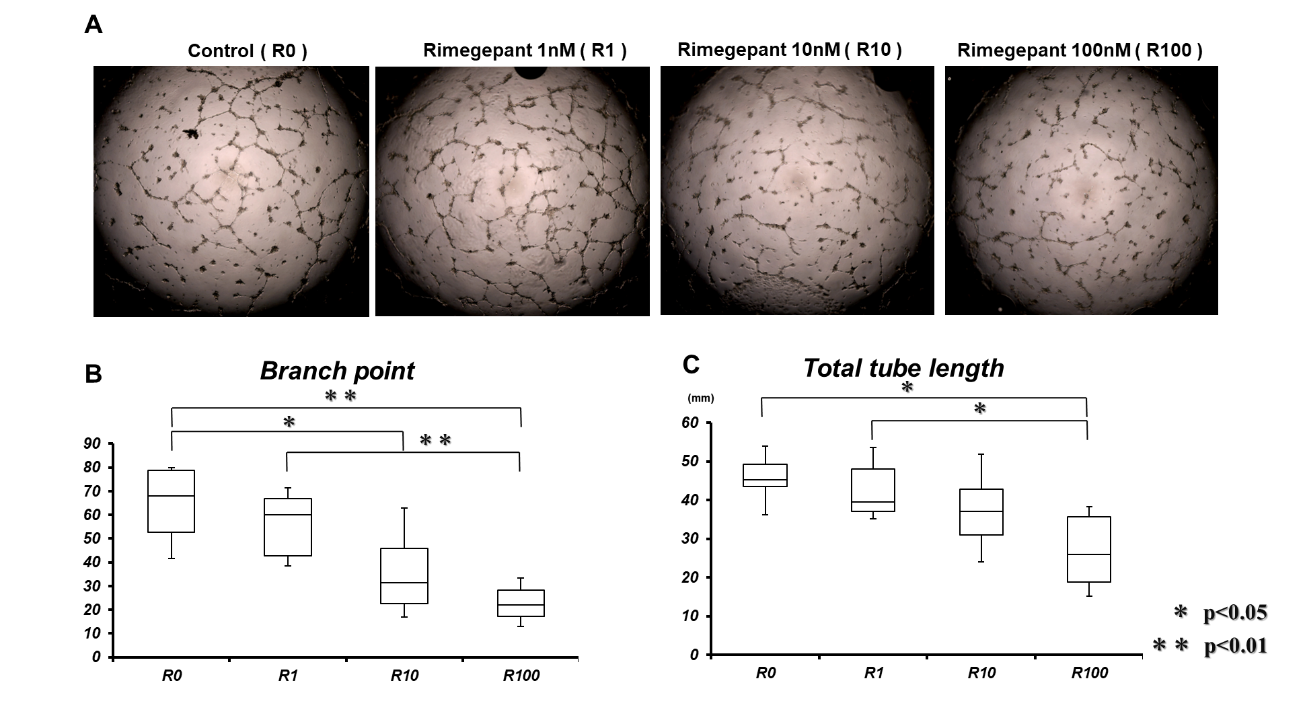


**Figure.2** (A) Tube formation of human umbilical endothelial cell (HUVECs) at 8 h. (B) The branch points of HUVECs. (C) The total length of the HUVECs.

**Reference**

1. Nagira K, Ikuta Y, Shinohara M, Sanada Y, Omoto T, Kanaya H, Nakasa T, Ishikawa M, Adachi N, Miyaki S *et al*: **Histological scoring system for subchondral bone changes in murine models of joint aging and osteoarthritis**. *Sci Rep* 2020, **10**(1):10077.

2. Sanada Y, Ikuta Y, Ding C, Shinohara M, Yimiti D, Ishitobi H, Nagira K, Lee M, Akimoto T, Shibata S *et al*: **Senescence-accelerated mice prone 8 (SAMP8) in male as a spontaneous osteoarthritis model**. *Arthritis Research & Therapy* 2022, **24**(1):235.

3. Gholobova D, Decroix L, Van Muylder V, Desender L, Gerard M, Carpentier G, Vandenburgh H, Thorrez L: **Endothelial Network Formation Within Human Tissue-Engineered Skeletal Muscle**. *Tissue Eng Part A* 2015, **21**(19-20):2548-2558.
